# The crippling grip of antimicrobial resistance in dentistry: A review

**DOI:** 10.6026/973206300211871

**Published:** 2025-07-31

**Authors:** Ena Sharma, Simran Thind, Tanvi Ohri, Radhika Goyal, Rajan Dhawan, Jotsaroop Singh, Sreejith Krishna, Ruhee Sangha

**Affiliations:** 1 Department of Periodontic, Oral Medicine and Radiology, Pedodontics, Prosthodontics, Rayat Bahra Dental College and Hospital, Mohali, Punjab, India; 2 Department of Periodontics and Endodontics, Maharish Markandeshwar College of Dental Sciences and Research, Mullana, Haryana, India

**Keywords:** Antimicrobial sensitivity, antimicrobial stewardship program, antimicrobial resistance infectious diseases, resistance to antimicrobial drugs, antimicrobial resistance

## Abstract

A primary challenge for dentists is maintaining or restoring a balanced oral microbiome, as it plays a significant role in determining oral health and disease states. However, oral diseases such as gingivitis, dental caries, periodontitis and peri-implantitis can occur when pathogenic microbes settle in the oral cavity and become part of the oral biofilm. There are several preventative and therapeutic strategies available today, but the majority of them are centered on antibiotics. Antibiotic stewardship in dentistry might be a helpful strategy to maximize and prevent inappropriate or even needless antibiotic usage, marking a step towards precision medicine, given the current context of antimicrobial resistance (AMR). Additionally, efforts are being made to discover novel, efficient treatments that can take the place of antibiotics.

## Background:

Antimicrobial resistance (AMR) in dentistry has emerged as a critical public health concern. The effectiveness of antibiotics, once a cornerstone of oral healthcare, is increasingly challenged by the evolution of resistant bacterial strains [1]. This review explores the current state of AMR in dentistry, focusing on its prevalence, contributing factors, clinical impact and potential solutions. The once-reliable shield of antibiotics in oral healthcare is facing a formidable foe: antimicrobial resistance (AMR). This growing public health concern threatens the effectiveness of these life-saving drugs, jeopardizing our ability to combat infections that were once readily treatable. From routine procedures like root canals to more complex surgeries, dentists have long relied on antibiotics to prevent and manage bacterial complications. However, the misuse and overuse of antibiotics have fuelled the emergence of resistant bacterial strains, rendering many antibiotics ineffective against these evolved pathogens [2]. This review delves into the current landscape of AMR in dentistry, uncovering its concerning prevalence, the factors driving its rise and the significant clinical impact it poses. Therefore, it is of interest to report potential solutions to combat this growing threat, emphasizing the need for both individual and collective action to preserve the efficacy of antibiotics in oral healthcare and ensure optimal patient outcomes.

## Prevalence of AMR in oral bacteria:

## Key findings and implications:

Antimicrobial resistance (AMR) among oral bacteria has become a growing concern in dental and public health communities. Key oral pathogens such as *Streptococcus mutans*, *Enterococcus faecalis*, *Porphyromonas gingivalis* and *Aggregatibacter actinomycetemcomitans* are increasingly showing resistance to commonly prescribed antibiotics like amoxicillin, metronidazole, tetracyclines and macrolides. Multidrug resistance is particularly evident in *E. faecalis*, especially in endodontic failures. The oral cavity serves as a significant reservoir for resistance genes due to the dense bacterial biofilms that facilitate horizontal gene transfer. Additionally, the overuse and misuse of antibiotics in routine dental procedures-often prescribed for self-limiting infections-further fuel the rise of resistant strains. Regional data from countries such as India and Brazil reveal alarmingly high rates of AMR, underscoring the global nature of this issue [3]. The implications of AMR in oral health are serious and far-reaching. Clinically, it leads to treatment failures, prolonged infections and complications following oral surgeries. From a broader perspective, resistant oral pathogens pose a systemic threat, as they can spread to other body sites and cause severe infections like endocarditis or sepsis [4]. Addressing this crisis requires the implementation of strict antimicrobial stewardship programs in dental practice, guided by evidence-based prescribing and continuous professional education. Furthermore, global health organizations emphasize the need for nationwide AMR surveillance in dentistry and support research into alternative therapies, such as probiotics, phage therapy and herbal antimicrobials. Without coordinated action, AMR in oral bacteria may severely undermine the effectiveness of routine dental care.

## Contributing factor to the rise of AMR in dentistry:

Several factors contribute to the rise of antimicrobial resistance (AMR) in dentistry, many of which stem from inappropriate prescribing practices and lack of awareness. One of the primary contributors is the overprescription of antibiotics for dental conditions that may not require them, such as irreversible pulpitis, routine extractions, or localized periodontal infections that can be managed by operative treatment alone [5]. Dentists may also prescribe antibiotics as a precaution rather than based on clinical evidence, often due to patient expectations or time constraints. Furthermore, the lack of adherence to antibiotic prescribing guidelines and insufficient knowledge about emerging resistance patterns among dental professionals exacerbate the problem [6]. Another major factor is the use of broad-spectrum antibiotics without culture sensitivity testing, especially in cases of endodontic or periodontal infections. This promotes the selection of resistant strains. Additionally, poor infection control practices in some dental clinics, along with the global rise in self-medication and over-the-counter availability of antibiotics, especially in developing countries, further accelerates resistance. The dense microbial environment of the oral cavity, particularly within dental biofilms, provides ideal conditions for horizontal gene transfer between bacteria, facilitating the spread of resistance genes. All these factors combined contribute significantly to the emergence and spread of AMR in dental settings [7].

## Strategies to control antimicrobial resistance (AMR) in dentistry:

Controlling antimicrobial resistance (AMR) in dentistry requires a multifaceted approach that focuses on prevention, education and responsible antibiotic use. One of the most effective strategies is the implementation of antimicrobial stewardship programs in dental settings, which promote the judicious use of antibiotics based on clinical guidelines and evidence-based protocols. Dentists should prescribe antibiotics only when clearly indicated, such as for spreading infections with systemic involvement and avoid prescribing them for conditions that can be managed through local dental treatment. Regular continuing dental education (CDE) programs on AMR and updated prescribing guidelines can significantly improve practitioners' awareness and prescribing habits [8]. Another key strategy is to enhance infection control and preventive care, including promoting oral hygiene, early diagnosis and routine dental check-ups to reduce the need for antibiotics. The use of narrow-spectrum antibiotics, when appropriate, along with culture and sensitivity testing in recurrent or severe infections can help tailor therapy and prevent resistance. Public education campaigns can discourage self-medication and over-the-counter access to antibiotics. Lastly, surveillance systems to monitor resistance trends in oral bacteria, along with research into alternative therapies like probiotics, antimicrobial peptides and photodynamic therapy, are essential to develop long-term solutions to combat AMR in dentistry [9].

## Antibiotic stewardship programs (ASPs) implemented in dentistry to combat antimicrobial resistance (AMR):

Antibiotic Stewardship Programs (ASPs) in dentistry are structured initiatives aimed at optimizing the use of antibiotics to combat the growing threat of antimicrobial resistance (AMR). These programs emphasize evidence-based prescribing, patient safety and preserving the efficacy of existing antibiotics. In dental practice, ASPs involve creating clinical guidelines for antibiotic use, training dental professionals to follow rational prescribing protocols and encouraging the use of antibiotics only when absolutely necessary-for instance, in cases of systemic involvement, cellulitis, or high-risk patients with specific medical conditions (*e.g.*, infective endocarditis prophylaxis) [10]. Key components of ASPs in dentistry include educational interventions, regular audit and feedback on prescribing practices and the integration of point-of-care decision support tools. National and international bodies such as the World Health Organization (WHO), Centers for Disease Control and Prevention (CDC) and FDI World Dental Federation have emphasized the importance of implementing ASPs in dental settings [10, 11]. In some countries like the UK and Australia, dental ASPs are already part of broader national antimicrobial strategies, with dentists receiving regular updates and access to prescribing resources such as the Dental Antimicrobial Stewardship Toolkit. These programs not only improve clinical outcomes but also reduce unnecessary antibiotic exposure and the spread of resistance within communities [12].

## Future directions in AMR research:

## Research efforts are crucial to further our understanding and combat AMR in dentistry:

The future of AMR research is a multi-pronged attack, aiming to combat the rise of resistant bacteria while developing alternative treatment strategies. One key direction involves improved surveillance of AMR patterns. This includes developing more rapid and cost-effective diagnostic tools to identify resistant bacteria and monitor their spread. Additionally, researchers are exploring the potential of artificial intelligence (AI) to analyze vast amounts of data on antibiotic resistance and predict emerging threats. Another exciting area is the development of novel antimicrobials. This encompasses exploring new classes of antibiotics that target different bacterial vulnerabilities [13]. Additionally, scientists are investigating bacteriophages (viruses that kill bacteria) and antimicrobial peptides as potential alternatives to traditional antibiotics. Research on repurposing existing drugs or combinations of drugs for new antimicrobial applications is also gaining traction. Finally, promoting responsible antibiotic use through education and stewardship programs remains crucial. This includes initiatives targeting both healthcare professionals and the public to ensure antibiotics are only used when truly necessary and at the appropriate dose and duration. By combining these diverse research directions, we can develop a more comprehensive strategy to combat the growing threat of AMR [14].

## Discussion:

The growing burden of antimicrobial resistance (AMR) in dentistry represents a silent but significant threat to both dental and general healthcare. As highlighted in the article "*The Crippling Grip of Antimicrobial Resistance in Dentistry*," the misuse and overuse of antibiotics in routine dental practice-often in the absence of clinical necessity-has been a key driver in the emergence of resistant oral pathogens. Dental practitioners, though prescribing a relatively small proportion of total antibiotics globally, are often found to deviate from established guidelines, prescribing antibiotics empirically for conditions like reversible pulpitis, localized abscesses and post-extraction pain where mechanical treatment would suffice [15]. This not only undermines the principles of rational antibiotic use but also facilitates the selection of multidrug-resistant strains within the oral microbiome [16]. The article rightly emphasizes the urgent need for integrating antibiotic stewardship into routine dental practice. Despite being preventable, AMR continues to rise due to a lack of awareness, inadequate surveillance and unrestricted access to over-the-counter antibiotics in many parts of the world. The oral cavity, with its complex and dense microbial biofilm, provides an ideal environment for horizontal gene transfer, allowing resistance genes to spread rapidly among commensals and pathogens [17]. To address this, the implementation of Antimicrobial Stewardship Programs (ASPs) specifically tailored for dentistry is crucial. These programs must focus on continuous education, monitoring of prescribing behaviors and public awareness. Furthermore, encouraging preventive dental care, promoting oral hygiene and investing in alternative antimicrobial therapies such as probiotics, antimicrobial peptides and photodynamic therapy could serve as sustainable long-term strategies [18]. As the article warns, without immediate and coordinated action, AMR may severely compromise the efficacy of dental care and increase the risk of systemic complications arising from otherwise manageable oral infections. The global fight against antimicrobial resistance (AMR) in healthcare is a complex and collaborative effort. The World Health Organization (WHO) spearheads this battle with its Global Action Plan on AMR, focusing on raising awareness, improving surveillance, reducing infection rates, optimizing antibiotic use and fostering research for new treatments. Additionally, organizations like the CDC promote antibiotic stewardship programs, while GARDP prioritizes developing and ensuring access to new antibiotics [19]. The ECDC monitors AMR through extensive surveillance networks. At the institutional level, hospitals implement antibiotic stewardship programs to monitor and optimize antibiotic use, reducing the emergence of resistant infections. Infection control committees enforce practices like hand hygiene, sterilization and isolation to prevent the spread of resistant bacteria. Surveillance systems track infection rates and resistance patterns, informing targeted interventions [20]. Research and development efforts in universities and medical centers aim to discover new antibiotics, alternative therapies like bacteriophages and advanced diagnostic tools. Educational programs train healthcare professionals in proper antibiotic prescribing, infection prevention and the importance of antimicrobial stewardship [21]. This multi-pronged approach is crucial for mitigating AMR in dentistry, where procedures can introduce bacteria into the bloodstream. By addressing AMR, we can improve patient outcomes, reduce complications and preserve the effectiveness of life-saving antibiotics for future generations.

## Conclusion:

Antimicrobial resistance in dentistry is a complex and evolving public health challenge. Understanding its prevalence, contributing factors and clinical impact is crucial for developing effective strategies to combat this growing threat. By promoting responsible antibiotic use, developing rapid diagnostics, AMR is a growing threat in dentistry with serious implications for patient care. By implementing antibiotic stewardship programs, exploring alternative therapies and fostering global collaboration, the dental community can play a vital role in mitigating the rise of AMR and safeguarding the effectiveness of antibiotics for future generations.
